# The Host Cell Receptors for Measles Virus and Their Interaction with the Viral Hemagglutinin (H) Protein

**DOI:** 10.3390/v8090250

**Published:** 2016-09-20

**Authors:** Liang-Tzung Lin, Christopher D. Richardson

**Affiliations:** 1 Department of Microbiology and Immunology, School of Medicine, College of Medicine, Taipei Medical University, Taipei 11031, Taiwan; ltlin@tmu.edu.tw; 2 Graduate Institute of Medical Sciences, College of Medicine, Taipei Medical University, Taipei 11031, Taiwan; 3 Department of Microbiology and Immunology, Dalhousie University, 5850 College St., Halifax, NS B3H 4R2, Canada; 4 Department of Pediatrics and Canadian Center for Vaccinology, Izaak Walton Killam Health Centre, Halifax, NS B3K 6R8, Canada

**Keywords:** measles virus, membrane cofactor protein, CD46, signaling lymphocyte activation molecule family member 1, SLAMF1, SLAM, CD150, nectin-4, polio virus receptor like protein 4, PVRL4

## Abstract

The hemagglutinin (H) protein of measles virus (MeV) interacts with a cellular receptor which constitutes the initial stage of infection. Binding of H to this host cell receptor subsequently triggers the F protein to activate fusion between virus and host plasma membranes. The search for MeV receptors began with vaccine/laboratory virus strains and evolved to more relevant receptors used by wild-type MeV. Vaccine or laboratory strains of measles virus have been adapted to grow in common cell lines such as Vero and HeLa cells, and were found to use membrane cofactor protein (CD46) as a receptor. CD46 is a regulator that normally prevents cells from complement-mediated self-destruction, and is found on the surface of all human cells, with the exception of erythrocytes. Mutations in the H protein, which occur during adaptation and allow the virus to use CD46 as a receptor, have been identified. Wild-type isolates of measles virus cannot use the CD46 receptor. However, both vaccine/laboratory and wild-type strains can use an immune cell receptor called signaling lymphocyte activation molecule family member 1 (SLAMF1; also called CD150) and a recently discovered epithelial receptor known as Nectin-4. SLAMF1 is found on activated B, T, dendritic, and monocyte cells, and is the initial target for infections by measles virus. Nectin-4 is an adherens junction protein found at the basal surfaces of many polarized epithelial cells, including those of the airways. It is also over-expressed on the apical and basal surfaces of many adenocarcinomas, and is a cancer marker for metastasis and tumor survival. Nectin-4 is a secondary exit receptor which allows measles virus to replicate and amplify in the airways, where the virus is expelled from the body in aerosol droplets. The amino acid residues of H protein that are involved in binding to each of the receptors have been identified through X-ray crystallography and site-specific mutagenesis. Recombinant measles “blind” to each of these receptors have been constructed, allowing the virus to selectively infect receptor specific cell lines. Finally, the observations that SLAMF1 is found on lymphomas and that Nectin-4 is expressed on the cell surfaces of many adenocarcinomas highlight the potential of measles virus for oncolytic therapy. Although CD46 is also upregulated on many tumors, it is less useful as a target for cancer therapy, since normal human cells express this protein on their surfaces.

## 1. Introduction

Measles virus (MeV) is a paramyxovirus that contains a 15 kilobase non-segmented RNA genome encoding nucleocapsid protein (NP), phosphoprotein (P), virulence factors (C and V), matrix protein (M), membrane fusion protein (F), hemagglutinin (H), and an RNA dependent RNA polymerase (L) [1,2,3,4]. The virus possesses a membrane envelope which contains the two viral glycoproteins, H and F. H protein mediates attachment to the host cell receptor, while F directs fusion of the viral envelope with host plasma membrane and syncytia formation, leading to cytopathic effects and cell death. The reverse genetics system developed by Martin Billeter is used by many laboratories to engineer the virus [5,6]. In this process, positive sense cDNA template under control of T7 promoter is transfected into 293 HEK cells along with expression vectors encoding the NP, P, L, and T7 polymerase proteins. The system has been used to introduce enhanced green fluorescent protein (eGFP) and firefly luciferase (Luc) into MeV and enables one to track virus dissemination during infections [7,8]. Binding of the H protein to a cellular receptor is the initial event of infection. This attachment of H to its host’s receptor triggers the F protein to generate fusion between the virus and the host cell membrane [9,10,11,12,13]. The quest for MeV cellular receptors began with vaccine/laboratory virus strains and progressed to more relevant receptors used by wild-type MeV (wtMeV) (reviewed in [14]).

## 2. The Hemagglutinin Protein and Its Properties

**Figure 1:**
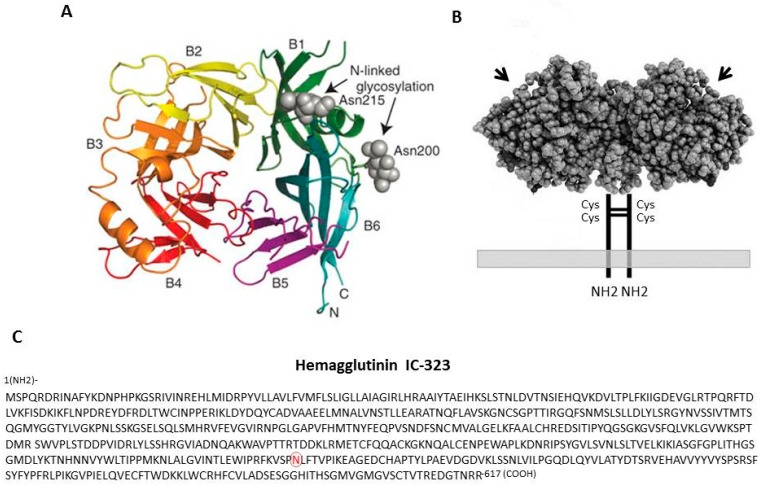
Structure of the head region from the hemagglutinin (H) protein of measles virus (MeV). (**A**) View from the top of the H protein showing the six β-sheet regions of the propeller-like structure; (**B**) View of the H protein from the side showing the dimer formed through two cysteine linkages in the stem region. Arrows indicate central head region; (**C**) Amino acid sequence of the H protein from the IC-323 strain of MeV. The N481 (red) residue is mutated to Y481 in vaccine/laboratory strains of MeV, enabling H protein to bind to the CD46 receptor. Panel A is adapted with permission from the Nature Publishing Group, Macmillan Publishers Ltd: Colf, L.M.; Juo, Z.S.; Garcia, K.C. *Nat. Struct. Mol. Biol* **2007**, *14* 1227–1228 [43]; Panel B is adapted from the American Society of Microbiology Journals: Rasbach, A.; Abel, T.; Münch, R.C.; Boller, K.; Schneider-Schaulies, J.; Buchholz, C.J. *J. Virol.* **2013**, *87*, 6246–6255 [47].

Humans and some monkey species are hosts for MeV [15,16,17,18,19,20,21], while rodents are not normally infected by the virus [1,22,23,24,25]. MeV isolates were originally isolated and adapted to grow in laboratory primate cell lines (reviewed in [26]). In 1954, John Enders isolated a strain of measles virus from a young patient named David Edmonston using human kidney cells [27]. It was later adapted to continuous monkey kidney cell lines (Vero and CV-1), but the virus did not readily grow in non-primate cell lines. However, this virus was eventually passaged in chick embryo fibroblasts to yield the first measles attenuated virus vaccine [28,29,30,31,32]. Tissue-culture-cell-adapted virus isolates were found to agglutinate the red blood cells of most old world monkeys but not those of humans [33,34,35,36]. The laboratory-adapted Edmonston strain was primarily used to deduce the molecular properties of measles virus and the hemagglutination properties were assigned to one of the viral membrane glycoproteins. This surface glycoprotein from the adapted virus was named hemagglutinin (H) protein. The gene for H protein was first cloned in 1986 and its deduced sequence was reported [37]. Sequences for other vaccine and wild-type isolates were reported by the Center for Disease Control (CDC) [38,39,40]. It is a 617 amino acid type II glycoprotein anchored by a single membrane spanning region near its amino terminus. H protein from the Edmonston strain of MeV contains five potential N-linked glycosylation sites between amino acids 168 and 238 while H proteins from recent wild-type isolates have a sixth glycosylation site at amino acid 416. Glycosylation mediates folding and export of the glycoprotein. During the late 1990s, it became apparent that it was more relevant to study wild-type isolates of MeV and it has been observed that mutations including N481Y and E451V occur during the process of adaptation in cell culture [41,42]. These mutations confer the ability of the laboratory/vaccine strains to use CD46 as a receptor, whereas wild-type isolates do not. Structural studies using X-ray crystallography demonstrated that H protein consists of an α-helical stalk supporting a cubic-shaped six-blade β-propeller head structure [9,43]. Each of the blade domains contains four antiparallel β-strands connected sequentially through extended loops (Figure 1). The H protein of MeV forms disulfide-linked dimers on the viral surface that in turn associate to form tetramers [44]. H protein acts in association with F protein to activate fusion during entry and cell-to-cell transmission [13,45]. The fusion activity is associated with conformational changes in F protein that are triggered as H binds to its receptors (reviewed in [46]).

## 3. The Membrane Cofactor Protein CD46 Is a Cellular Receptor for the Hemagglutinin Proteins of Laboratory-Adapted and Vaccine Strains of Measles Virus

**Figure 2:**
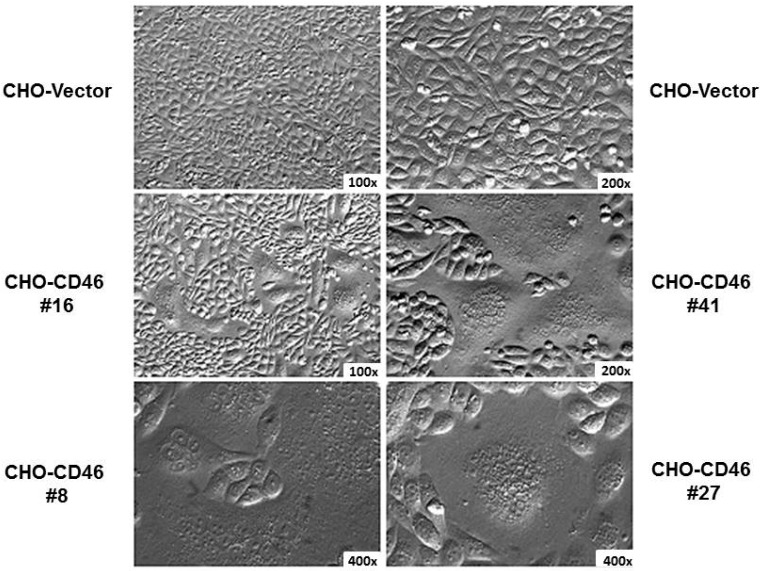
Chinese hamster ovary (CHO) and CHO-CD46 cells infected for 48 h with the Edmonston vaccine strain of MeV. The CD46 coding region (BC2 isoform) was expressed using a dihydrofolate reductase (DHFR) amplification vector under control of the cytomegalovirus (CMV) promoter. Four different cell lines (#8, #16, #27, #41) are shown at indicated magnifications (100×, 200×, or 400×) using Nomarsky optical microscopy. Cells were infected at a multiplicity of infection (m.o.i.) of 1. Syncitia/multinucleated cells were clearly apparent in the infected cells at 48 h post-infection.

**Figure 3:**
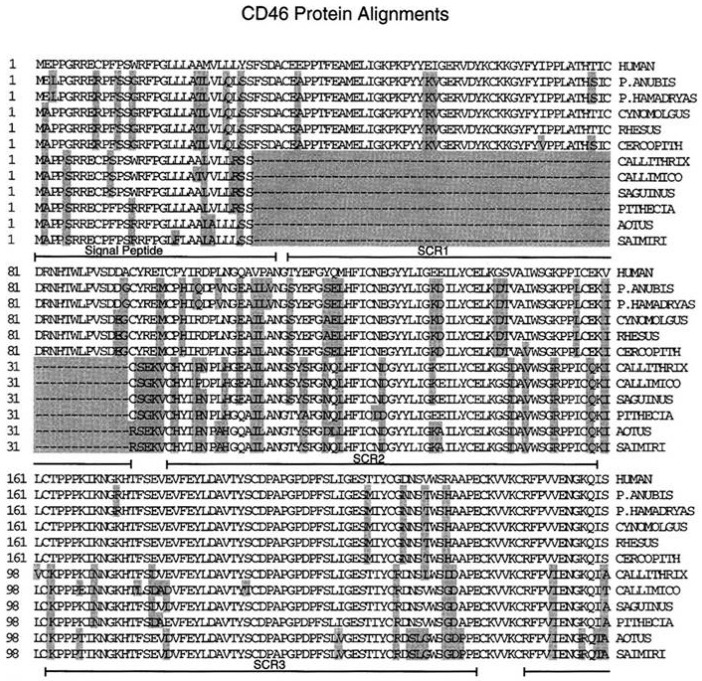
Alignment of CD46 proteins derived from complementary DNAs (cDNAs) prepared from the lymphocytes of humans, Old World, and New World monkeys. CD46 molecules from New World monkeys contain a deletion of the short consensus repeat 1 (SCR1) domain due to alternative messenger RNA (mRNA) splicing. Shaded residues indicate amino acids that differ from the human sequence. Baboons (*Papio anubis*, *Papio hamadryas*), macaques (rhesus monkey, cynomolgus monkey), African green monkey (*Cercopithecus aethiops*), marmosets (*Callithrix jaccus*, *Callimico goeldii*, *Saguinus oedipus*), saki (*Pithecia pithecia*), owl monkey (*Aotidae aotus*), squirrel monkey (*Saimiri sciureus*). Reprinted with permission from the American Society of Microbiology Journals: Hsu, E.C.; Dörig, R.E., Sarangi, F.; Marcil, A; Iorio, C.; Richardson, C.D. *J. Virol.* **1997**, *71*, 6144–6154 [58].

Natural isolates of MeV can be rapidly adapted to grow in Vero monkey kidney cells, and the Edmonston virus has been propagated in these cells for many years. Laboratory-adapted measles strains infect a wide variety of human and monkey cell lines derived from different organs including lung (HEL), kidney (Vero, CV-1, HEK 293), bladder, prostate, and cervix (HeLa). We employed a genetic approach to identify the receptor for Edmonston MeV [48,49]. Mouse and hamster cells could not support infections by the Edmonston strain of MeV and we also demonstrated that rodent cell lines could not bind the virus [48]. Mouse–human hybrid cell lines were tested for their ability to bind MeV using a monkey red blood cell (MRBC) rosette assay. Hybrid cell lines that contained an intact human chromosome 1 arm q32 were capable of binding virus, while cell lines with only part of chromosome 1 arm p could not [48,49]. An earlier publication from Gerlier’s group described a monoclonal antibody that blocked MeV infection and reacted with two polypeptides of molecular masses 57 kDa and 67 kDa [50]. Based upon all these criteria, a search through an encyclopedia of determinants for cell surface markers [51], and flow cytometry analysis with commercial antisera, indicated that the complement binding protein known as membrane complex protein (MCP) or CD46 [52,53] was a likely candidate for the MeV receptor. We subsequently proved this hypothesis by generating Chinese hamster ovary (CHO) cell lines that expressed human CD46 (Figure 2). Cells expressing this marker bound MRBC in the presence of MeV, and were susceptible to infection by the Edmonston strain of virus, forming syncytia and synthesizing viral proteins. An identical and independent conclusion was simultaneously published the same month by Gerlier’s group using the Halle strain of MeV [54]. They used their MeV neutralizing monoclonal antibody to immunoprecipitate the putative receptor and identified the protein by mass spectrometry. CD46 normally binds complement components C3b and C4b on a target cell and serves to protect homologous host tissue or sperm from complement mediated damage [52,53]. The MeV binding site on CD46 was mapped using mutagenesis by several laboratories, including our own, to the short consensus region 1 (SCR1) and short consensus region 2 (SCR2) domains [41,55,56,57,58,59,60,61,62,63,64,65,66]. In a study with blood samples from eight species of New World monkeys, we were able to show that erythrocytes from New World monkey were unable to hemagglutinate in the presence of Edmonston MeV [58]. Lymphocytes and kidney cell lines from these New World monkeys contained a major deletion of SCR1 in their CD46 molecules due to a difference in messenger RNA (mRNA) splicing (Figure 3). Subsequently, NZP60 and OMK cell lines derived from common marmoset and owl monkeys, respectively, were also shown to contain the deleted form of CD46, and could not be infected with laboratory strains of MeV [58]. The SCR1 deletion was confirmed by John Atkinson’s group for most cells and tissues of marmoset and squirrel monkeys. However, they did find the undeleted isoform of CD46 on spermatozoa [67]. The evolutionary and clinical significance for this SCR1 deletion in different types of monkeys is still unclear. It may be more relevant to other pathogens such as *Neisseria meningitidis* [68], human herpes virus 6 [69], adenovirus (groups B and D) [70,71], and bovine diarrhea virus, which also use CD46 as a receptor [72].

**Figure 4:**
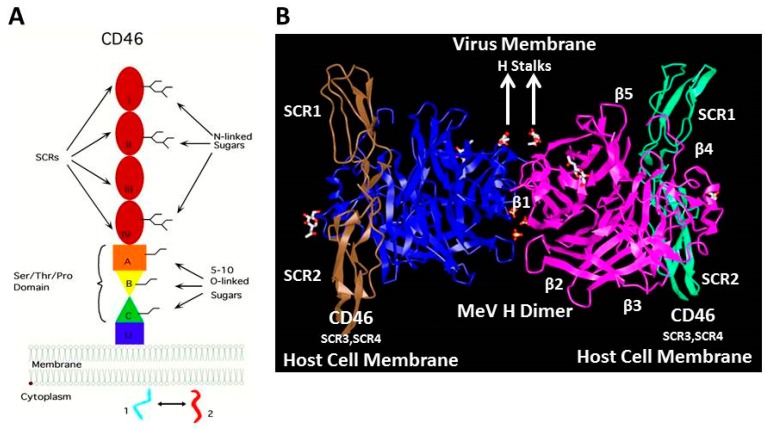
Interaction of CD46 with H dimer from the vaccine strain of MeV. (**A**) Schematic of membrane cofactor protein (MCP) or CD46. Protein is comprised of four short conserved regions (SCR1-SCR4), the Ser/Thr/Pro (STP) domain, transmembrane region, and two alternatively spliced cytoplasmic tails. MeV binds to SCR1 and SCR2 and complement components C3b, and C4b bind to SCR3 and SCR4. Sugars in SCR2 are important for MeV binding; (**B**) Structure of SCR1 and SCR2 domains of CD46 bound to H protein dimer head region. Adapted by permission from the Nature Publishing Group, Macmillan Publishers Ltd.: Santiago, C.; Celma, M.L.; Stehle, T.; Casasnovas, J.M. *Nat. Struct. Mol. Biol*. **2010**, *17*, 124–129 [73].

Structural analysis of the globular head of H protein complexed to SCR1 and SCR2 domains of CD46 revealed the interactions between the two proteins [73,74]. The crystal unit contained an H dimer bound to two CD46 molecules (Figure 4). A unique groove on the side of the β-propeller 4 domain of H engages residues in the SCR1 and SCR2 domains. Contact region 1 involves a Pro-Pro motif at the CD46 N-terminus that penetrates deep into a hydrophobic hole in the H protein. Important SCR1 residues for this interaction include Ile37, Pro38, Pro39, and Leu40 which form a plug that penetrates into the hydrophobic socket in H at the interface between blades β4 and β5. This protrusion is situated between the side chains of Leu464 and Leu500 in β4, and Tyr541 and Tyr543 of β5. Contact region 2 consists of the residues connecting SCR1 and SCR2. The Tyr481 hydroxyl of H forms a hydrogen bond with the carbonyl of Cys65 of CD46, and Gly546 of H introduces flexibility for high affinity binding. Contact region 3 involves almost the entire side of SCR2 where Tyr67 of CD46 aligns against Val451 of the H protein. In addition, the side chain of Tyr83 interacts with Tyr481 of H. The carbohydrate attached to Asn80 interacts with MeV H residue Lys488. The affinity of interaction between H from Edmonston MeV and CD46 was measured (*K_d_* = 79 nM) [75].

Following the discovery of CD46 as a receptor for laboratory strains of measles virus, several investigators developed CD46 transgenic mice to study the pathogenesis of MeV in a small animal model. Mice, rats, and guinea pigs do not express a CD46 homologue on their tissues, although they do have a CD46-like molecule on their spermatozoa [76]. Different promoters have been used to elicit tissue specific expression of CD46 in mice of several genetic backgrounds. The first CD46 transgenic mice were produced using the hydroxymethylglutaryl CoA reductase (HMGCR) promoter which drives ubiquitous expression in most tissues [77,78]. Although MeV replication was limited in normal tissues with functioning innate immunity, intracranial infection of newborn transgenic mice with Edmonston MeV produced severe neurological disease culminating in death. Infectious viral particles were produced, and the pathology was similar to measles encephalitis in immunocompromised patients. In another model designed to study MeV persistent brain infections, CD46 was expressed under control of the neuron specific enolase (NSE) promoter [79]. Following intracranial injection of Edmonston MeV, the NSE-CD46 neonates developed severe neurological disease and died, while adults were relatively resistant to infection. Disease was accompanied by inflammation and cytokine production in the brain. The complete gene of CD46 was also introduced into mice by two different laboratories. In the first, a yeast chromosome (YAC) containing the complete human CD46 gene was introduced into the mouse genome [80], and the various isoforms of CD46 were synthesized. Edmonston MeV was lethal following intracranial injection. YAC-CD46 mice were crossed into different immunodeficient backgrounds (recombination activating gene 1 (RAG-1) knockout (KO), CD4-KO, CD8-KO, breakpoint cluster region protein (BCR)-KO, Perforin-KO, tumor necrosis factor (TNF)-α KO, interferon (IFN)-γ KO) [81] and it was found that the interferon response and downstream adaptive immunity were critical in controlling infections. Similar experiments were performed by Cattaneo’s group using a full-length CD46 genomic clone in a mouse lacking the interferon alpha/beta receptor (IFNAR-KO) [82]. Following intranasal inoculation, virus infection was evident in the lungs, peripheral blood mononuclear cells (PBMCs), spleen, liver, and macrophages. Infections in all the different CD46 transgenic mouse lines were limited, since the animals were resistant to wtMeV isolates, and they required suppression of innate immunity to yield viable infections.

## 4. SLAMF1/SLAM/CD150 Is the Lymphocyte Cellular Receptor for the Hemagglutinin Proteins of Wild-Type and Vaccine Strains of Measles Virus

**Figure 5:**
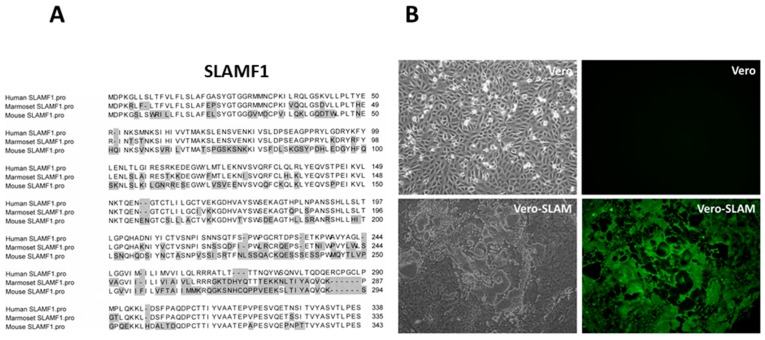
Wild type MeV can infect Vero cells that express human and marmoset SLAMF1 but not mouse SLAMF1. (**A**) Alignment of protein sequences of signaling lymphocyte activation molecule family member 1 (SLAMF1) from human (AAH12602.1), marmoset (XP_002760222), and mouse (AA17100.1) homologues. Shaded residues indicate amino acids that differ from the human sequence. Shaded residues indicate amino acids that differ from the human sequence. There is a high level of conservation between human and marmoset SLAMF1 sequences; (**B**) Vero-SLAMF1 cells can be infected with wild-type IC-323 strain of MeV that expresses the enhanced green fluorescent protein (eGFP) reporter gene. Cells were infected with virus at an m.o.i. of 5 for a period of 60 h. Syncytia were visible as early as 18 h.

We, and others, hypothesized that an alternative receptor to CD46 could be used by wtMeV [41,83,84,85]. Kobune’s group had shown that wtMeV could be isolated in B95-8 cells, a marmoset B cell line immortalized with Epstein Barr virus (EBV). The wtMeV grew in B95-8 cells without the need for adapting the virus to CD46 receptor usage [86]. These wtMeV isolates do not possess the ability to down-regulate surface expression of CD46 or hemagglutinate African green monkey red blood cells (RBCs), while laboratory strains that were adapted to Vero monkey kidney cells do [26,87,88]. In addition, CD46 could not serve as a receptor for MeV in tamarins or marmosets, since the SCR1 domain is deleted in the cells/tissues of New World monkeys through alternative splicing (Figure 3) [58,67]. Despite the SCR1 deletion, South American monkeys are still susceptible to laboratory strains and wtMeV infections, and the disease is primarily lymphotropic leading to secondary infections that cause gastroenterocolitis, pneumonitis, and bacteremia [1,15,16]. We subsequently provided indisputable evidence for the existence of a second receptor on activated lymphocytes. MeV that had been isolated in marmoset B95-8 cells bound to activated B lymphocytes and lymphomas [58]. A single tyrosine residue at amino acid 481 of H determined high affinity binding to CD46, but binding to the putative lymphocyte receptor was favored when asparagine was at this position [41]. The N481 residue was observed in all wild-type isolates grown in B95-8 cells, and could be converted to the Y481, to yield the CD46-binding phenotype, by passaging the virus 2–5 times in HeLa cells. As one would anticipate, polyclonal antibodies directed against CD46 did not inhibit infections of activated B cells with the wild-type virus [41]. Yusuke Yanagi’s group was first to identify signalling lymphocyte activation molecule family member 1 (SLAMF1/SLAM/CD150) as the lymphocyte receptor for MeV [89]. They used pools of cDNA expression clones derived from B95-8 lymphocytes which express SLAMF1, but not a functional CD46 receptor. This group transfected the cDNAs into 293T cells, which do not express SLAMF1, to screen for infectivity by vesicular stomatitis virus (VSV)-wtH,F pseudotypes. The pseudotypes did not recognize CD46 since they contained wtH protein. Pools each containing 450 different clones were further subdivided until it was determined that an individual expression plasmid containing the coding sequence for SLAMF1 could promote wtMeV infections. This study subsequently demonstrated that CHO cells expressing SLAMF1 were susceptible to wtMEV infections. Our laboratory confirmed this discovery with a similar approach [85]. Using cDNA expression libraries prepared from B95-8 lymphocytes and screening transfected CHO cells for susceptibility to wtMeV infection, our laboratory used magnetic beads conjugated to a monoclonal antibody directed against the H protein to isolate infected cells. The episomal expression plasmid encoding SLAMF1 was extracted and rescued from these cells. Amino acid sequences of SLAMF1 for humans and marmosets are highly similar, but differ substantially from the mouse homologue (Figure 5A). CHO or Vero cells expressing SLAMF1 became susceptible to infections with wtMeV and used this receptor without the need to adapt and use CD46 (Figure 5B) [90].

The respective regions of H protein that interact with SLAMF1 and CD46 were identified by Cattaneo’s group and used to generate viruses defective in binding to one or the other receptor [91]. H protein amino acids F431, V451, Y481, and A527 were crucial for CD46 binding and residues Y529, D530, and R533 were important for binding to SLAMF1. Mutations in H could be used to create selective receptor blind viruses. Although SLAMF1 was originally identified in B cell lymphoma cell lines, the receptor has since been shown to be expressed on activated B, T, monocyte, and dendritic cells [51,92,93]. All these cell types can be infected with wtMeV. The SLAM family receptors (SLAMF1–SLAMF9) modulate a wide range of functions including myeloid cell and lymphocyte development, T and B cell responses to viruses, bacteria, and parasites. However, only SLAMF1 can function as a receptor for MeV. SLAMF1 is a costimulatory molecule of the immunoglobulin (Ig) super family that contains a variable (V) and a constant (C2) region [94,95,96]. The intracellular region of SLAMF1 associates with the SH2 domain-containing adaptor protein (SAP) or another adaptor protein, Ewing’s sarcoma-associated transcript 2 (EAT-2). In T cells, SAP helps recruit Src kinases (Fyn, Lck, Src) and regulatory phosphatases (Src homology region 2 domain-containing phosphatase-1 (SHP-1), shp1/2). Signal transduction may trigger T cell proliferation, IFN-γ production, cytotoxic responses, and stimulate B cell proliferation characteristic of a T_H2_ response. Mutations in SAP have been linked to X-linked lymphoproliferative disease [97,98,99,100]. This disease appears in the context of EBV infection. In the absence of a functional SAP, EBV-infected B cells are not cleared and massive B and T cell expansion occurs. In monocytes, SLAMF1 can enhance phagocytosis, cytokine production, and migration of myeloid cells to sites of inflammation. Through activation of the lectin receptor dendritic cell- specific intercellular adhesion molecule-3 grabbing non-integrin (DC-SIGN) on dendritic cells, MeV can increase cell surface expression of SLAMF1 [101]. SLAMF1 can also serve as a microbial sensor for gram negative bacteria (*Salmonella*, *Escherichia coli*) on macrophages.

**Figure 6:**
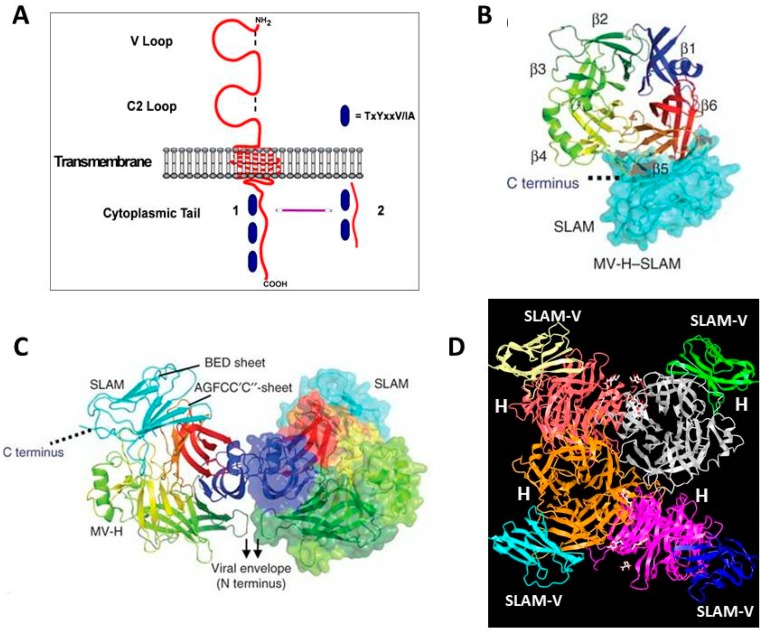
Structure of the head region from the H protein of MeV bound to the V region of SLAMF1. (**A**) Schematic of SLAMF1 showing the V and C2 regions of the extracellular domain, the membrane spanning region, and the intracellular region containing phosphotyrosine regions. Either of two cytoplasmic tails (1 or 2) may be present depending upon alternate splicing; (**B**) V region of SLAMF1 binding to β5 and β6 sheet regions from the head of the H protein viewed from the top; (**C**) Side view of V region of SLAMF1 binding to heads of the H dimer proteins; (**D**) V regions of four SLAMF1 molecules binding to the tetrameric H proteins. Panels B, C, and D adapted by permission from the Nature Publishing Group, Macmillan Publishers Ltd.: Hashiguchi, T.; Ose, T., Kubota, M.; Maita, N.; Kamishikiryo, J.; Maenaka, K.; Yanagi, Y. *Nat. Struct. Mol. Biol.* **2011**, *18*, 135–141 [102].

The published crystal structure of MeV H in complex with SLAMF1-V domain from Yanagi’s laboratory shows that β4–6 regions of H interact with the GFCC’C’’ region of the SLAMF1-V domain [101] (Figure 6). Salt bridges involving residues Asp530 and Arg 533 of H and Glu123 of SLAMF1-V play a major role in stabilizing the complex. An intermolecular β-sheet, comprised of residues Pro191–Arg196 (in β6) of H and residues Ser127–Phe131 of SLAMF1-V, further stabilizes the complex. The affinity of the MeV H and SLAMF1 interaction was measured (*K_d_* = 80 nM) [75]. The MeV H protein exists as a dimer of two disulfide linked H proteins in the viral membrane to form a tetramer structure which interacts with a trimeric F protein [44]. Crystallography revealed two conformational states of these tetrameric structures (Form I and Form II). Both conformations have identical binding interactions with SLAMF1-V. Hashiguchi et al. suggest that Form II has an important role in membrane fusion that follows receptor binding. They suggest that the shift of the MeV H tetramer could trigger the conformational change in F protein required for membrane fusion [102]. A similar change can also be envisaged with CD46 binding. The Cattaneo group proposed a different model where two head domains in a MeV H dimer twist relative to each other upon receptor binding to trigger membrane fusion [12].

**Figure 7:**
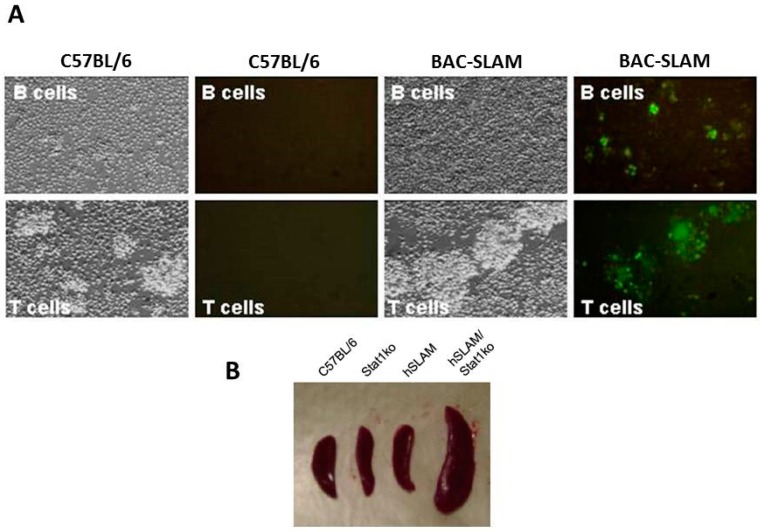
Infection of lymphocytes and spleen from human SLAMF1 transgenic mouse in Stat1(-/-) background with wtMeV expressing eGFP reporter protein. (**A**) T and B cells from infected mice express eGFP reporter protein; (**B**) Spleens from infected SLAMF1 transgenic mice in a Stat1(-/-) background are highly enlarged. Reprinted with permission from the Proc. Natl. Acad. Sci. U.S.A.: Welstead, G.G.; Iorio, C.; Draker, R.; Bayani, J.; Squire, J.; Vongpunsawad, S.; Cattaneo, R.; Richardson, C.D. *Proc. Natl. Acad. Sci. USA* **2005**, *102*, 16415–16420 [108].

Since mouse SLAMF1 differs considerably from the human homologue (Figure 5A), it cannot function as a receptor for MeV. A number of transgenic mouse models that express human SLAMF1 (hSLAMF1) have been developed using different promoters to drive the transgene. However, replication of MeV is still somewhat limited and many of the studies have been performed in mice with an immune deficient background. The first hSLAMF1 transgenic mouse was generated with the receptor under control of the Lck promoter, which is specific for T lymphocytes [103]. T cells, spleen lymphocytes, and thymocytes were susceptible to infection with wild-type and vaccine strains of MeV. Viral antigens were detected in the lymphocytes, although clinical symptoms were not observed. SLAMF1 transgenic mice were also generated using the CD11c promoter, which allows expression of the receptor in spleen and bone marrow derived dendritic cells [104]. Infection of dendritic cells (DCs) inhibited their function and caused MeV induced immune suppression in the mice [105]. DC cells that were infected inhibited T cell proliferation, and exhibited decreased synthesis of interleukin (IL)-12 following virus engagement of the Toll-like receptor 4 (TLR4) signal pathway [106]. Another laboratory expressed hSLAMF1 in mice under control of the ubiquitous HMGCR promoter, and suckling mice were highly susceptible to intranasal MeV infection [107]. Infected suckling mice developed severe neurological symptoms including lethargy, seizures, ataxia, and weight loss, and died within three weeks. More natural SLAMF1 transgenic models were generated by incorporating the entire *SLAMF1* gene under control of its native promoter. The first of these was performed in our own laboratory and incorporated the gene from a hSLAMF1 bacmid into the mouse genome [108]. hSLAMF1 was expressed in activated B, T, and dendritic cells. Limited and transient infection of nasal lymph nodes was observed in wild-type mice infected via the intranasal route with wtMeV-eGFP. To enhance infection, these SLAMF1 transgenic mice were crossed into a signal transducer and activator of transcription 1 (STAT-1) deficient background. After either intranasal or intraperitoneal inoculation, infection of the thymus, spleen, and lymph nodes was detected. Infected T and B cells, enlarged lymph nodes and splenomegaly were apparent (Figure 7). This same genomic hSLAMF1 mouse model was further investigated with mice having an IFNAR-KO background [109]. To identify the cells undergoing primary MeV infection, mice were inoculated intranasally with wtMeV-eGFP. One day after inoculation 2.5% of the alveolar macrophages and 0.5% of the dendritic cells in the airways were infected. Later, at 2–3 days post-infection, MeV infected B and T lymphocytes and virus could be isolated from lymphatic tissue including mandibular, mediastinal, mesenteric, and inguinal lymph nodes, and spleen. This model recapitulated early events in human MeV infections. Using mouse “knockin technology”, a refined model from Yanagi’s laboratory was generated by producing a chimeric SLAMF1 molecule in mice where the V domain of mouse SLAMF1 was replaced by that of human SLAMF1 [110]. Again, MeV could only grow in hSLAMF1 knockin mice that were bred on an IFNAR-KO background. Following intranasal or intraperitoneal inoculation, MeV was detected in the spleen and lymph nodes throughout the mice. A subsequent paper using this mouse showed that MeV infections caused lymphopenia, inhibition of T cell proliferation and antibody production, increased production of IL-4 and IL-10, and the suppression of contact hypersensitivity [111]. Indications were that this hSLAMF1 transgenic mouse is a useful small animal model for MeV-induced immunosuppression. Interestingly, the cotton rat has also been shown to be a semipermissive small animal model for MeV pathogenesis [112]. wtMeV replicates in the lymph nodes, spleen, and lung of this animal. Infection was shown to be due to wtMeV’s ability to use the cotton rat SLAMF1 as a receptor, unlike the mouse homologue [113]. However, the cotton rat SLAMF1’s function as a receptor was 10 times worse than the human homologue, and it produced smaller plaques and lower wtMeV titers in Vero-cotton rat SLAMF1 cells [113].

## 5. The Epithelial Cell Receptor for the Hemagglutinin Protein of Wild-Type Measles Virus

In vivo experiments with macaques indicated that alveolar macrophages, dendritic cells, and lymphocytes, which express SLAMF1, probably constituted the primary targets for measles virus infections [114,115,116,117]. However, wtMeV has been shown to infect the epithelial cells of the lungs, bronchial tubes, trachea, pharynx, esophagus, mouth, liver, intestines, and bladder at late times post-infection [118,119,120,121,122,123]. Epithelial cells do not express SLAMF1, but the infected cells do shed virus [1,123,124]. Consequently, epithelial cells were thought to be important later on in infection, and for the spread of MeV by aerosol droplets. In macaques infected with wtMeV-eGFP, the epithelial cells of the upper respiratory tract were infected at the peak of virus replication [114,125] and it has been reported that more virus is shed by tracheal cells than by lymphocytes, late in infection [115,123]. At late stages of human infection, MeV has been reported to cause extensive damage to upper respiratory tract and lung epithelial cells [125,126]. Giant cell pneumonia is a severe respiratory condition caused by MeV in individuals with compromised immune systems, and is characterized by multinucleated lung epithelial cells that contain MeV proteins and particles [127,128,129].

**Figure 8:**
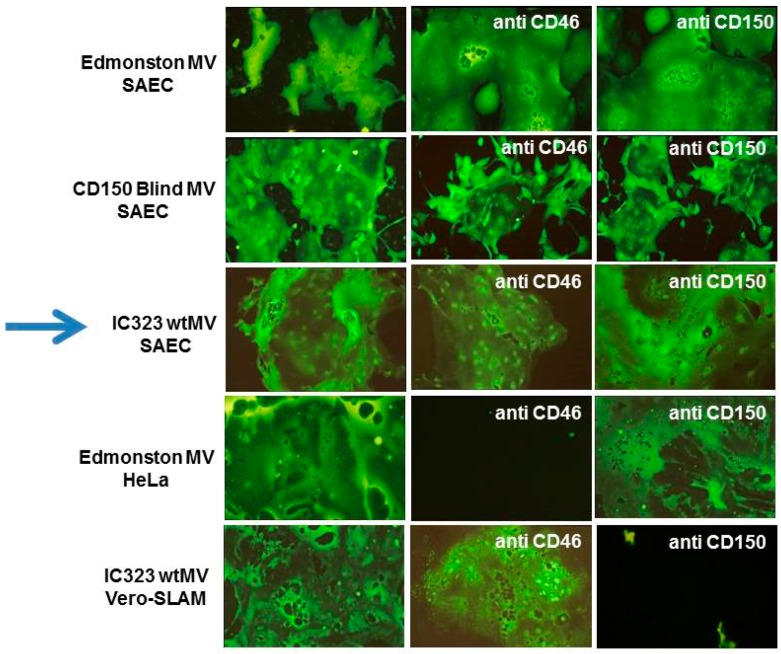
Small airway epithelial cells (SAECs) grown in 2% fetal calf serum can be infected with wild-type IC-323 measles virus in the absence of SLAMF1, and independently of CD46 receptor. Neutralizing antibodies against CD46 and SLAMF1 receptors have no effect upon infections of SAEC’s with Edmonston vaccine or wild type IC-323 measles virus (indicated by the arrow). Antibodies against CD46 inhibit infections of HeLa cells by the Edmonston vaccine MeV and antibodies against SLAMF1 block infections of Vero-SLAM cells with wild type IC-323 MeV. Reprinted by the author from PLoS Pathogens under the Creative Commons Attribution agreement from: Noyce, R.S.; Bondre, D.G.; Ha, M.N.; Lin, L.T.; Sisson, G.; Tsao, M.S.; Richardson, C.D. *PLoS Pathog*. **2011**, *7*, e1002240 [133].

Wild-type MeV does not readily infect laboratory fibroblast, endothelial, or epithelial cell lines and its spread to these cell types in infected macaques is limited [114,115,116]. However, Tashiro’s group first showed that wtMeV could readily infect primary human small airway epithelial cells (SAECs) if they were actively growing in 2% fetal calf serum [124]. These cells do not express SLAMF1 and wtMeV cannot use CD46. This finding suggested that there was another receptor on epithelial cells [124]. We confirmed and reported similar results at the Negative Strand Virus Meeting in Salamanca (2006) [130]. These cells did not express SLAMF1 and virus infections could not be blocked with antibodies against either SLAMF1 or CD46 (Figure 8). Marmoset SAECs which lack CD46 [58], as well as CD150, could also be infected with wtMeV in the presence of 2% fetal calf serum. These results strongly indicated that there was an additional receptor for MeV on airway epithelial cells. Subsequently, Yanagi’s laboratory discovered that the lung adenocarcinoma cell line NCI-H358 was susceptible to infection by wtMeV [131]. We also showed that many adenocarcinoma cell lines from the lung, breast, and colon could be readily infected with wtMeV (Negative Strand Virus Meeting in Brugge 2010; [132]). Again, these adenocarcinoma cell lines did not express SLAMF1 nor were wtMeV infections blocked by antibodies against MCP/CD46 or SLAMF1. The third receptor was hypothesized to lie on the basolateral side of polarized epithelial cells in close proximity to infected lymphocytes [115,116,133,134], but we also found the receptor/entry site is on the apical surface of adenocarcinoma cells [132,133]. Other investigators were also searching for this receptor on cancer cell lines. Through microarray analysis, it was determined that loss of tight junction proteins, caused by the expression of SNAIL transcription repressor, inhibited wtMeV infections [137]. Using site-specific mutagenesis, the H protein amino acids that interact with the putative epithelial receptor were mapped on the 3-D structure of H protein, and include residues I456, L464, L482, P497, Y541, and Y543 [115,135].

## 6. Identification of Nectin-4 (PVRL4) as the Epithelial Receptor for MeV

**Figure 9:**
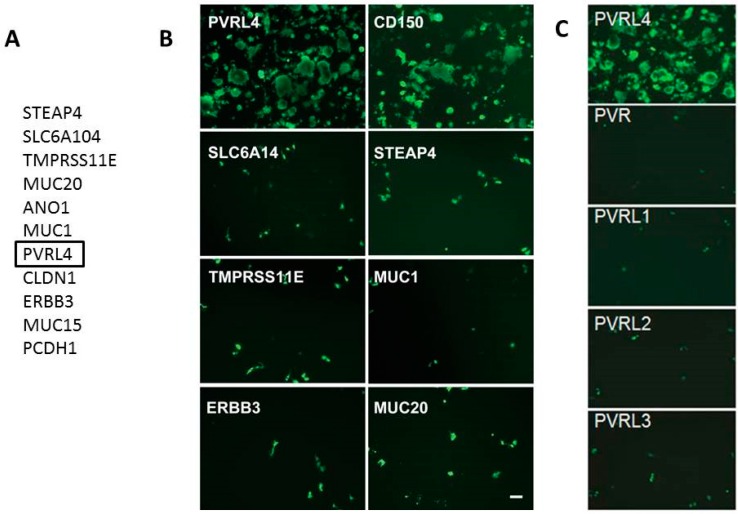
Microarray analysis reveals that Nectin-4 is a receptor for wild type MeV. (**A**) Comparative microarray analysis of mRNAs from permissive versus non-permissive cancer cell lines and SAECs grown in the presence or absence of 2% fetal calf serum revealed 11 candidate cellular receptors; (**B**) COS-1 monkey kidney cells were transfected with expression plasmids encoding PVRL4 (Nectin-4), CD150 (SLAMF1), solute carrier family 6 member 14 (SLC6A14), six transmembrane epithelial antigen of prostate 4 (STEAP4), transmembrane serine protease 11E (TMPRSS11E), mucin 1 (MUC1), erb-b2 receptor tyrosine kinase 3 (ERBB3), and mucin 20 (MUC20) genes. After 36 h, cells were infected with wild type IC-323 MeV expressing the eGFP reporter protein. Only PVRL4 (Nectin-4) and CD150 (SLAMF1) expression supported wtMeV infection of the COS-1 host cells. Fluorescence micrographs were taken at 36 h post-infection; (**C**) COS-1 cells were transfected with expression plasmids encoding PVRL4 (Nectin-4), poliovirus receptor (PVR), PVRL1 (Nectin-1), PVRL2 (Nectin-2), and PVRL3 (Nectin-3). Only PVRL4 (Nectin-4) expression supported wtMeV infection. Reprinted by the author from PLoS Pathogens under the Creative Commons Attribution agreement from: Noyce, R.S.; Bondre, D.G.; Ha, M.N.; Lin, L.T.; Sisson, G.; Tsao, M.S.; Richardson, C.D. *PLoS Pathog*. **2011**, *7*, e1002240 [133].

Nectin-4/PVRL4 was recently shown by our laboratory, and confirmed by Cattaneo’s group, to be the elusive epithelial receptor for MeV [133,138]. We showed that wtMeV infected primary airway epithelial cells grown in fetal calf serum, and many lung, breast, and colon adenocarcinoma cell lines. Adenocarcinomas are defined as tumors with glandular histology that arise from epithelial cells. The virus receptor appeared to be on the apical surface of permissive cancer cells, as well as the basolateral surface. Many cancer cell lines that were susceptible to wtMeV are polarized, yet disruption of adherens and tight junctions with phorbol esters had no effect upon viral infections. Transfection of several non-permissive cancer cell lines (MDA-MB-231 and A549 cells) with an expression vector encoding SLAMF1 rendered them susceptible to measles virus. This indicated that these adenocarcinoma cells were virus replication competent, but lacked a receptor needed for virus infection. Microarray analysis of permissive versus non-permissive cell lines, and a comparison of the mRNA transcripts for membrane proteins, led us to compile a list of 11 candidate receptors. Only the human tumor cell marker Nectin-4/PVRL4 made cells permissive to MeV (Figure 9). Flow cytometry confirmed that Nectin-4 was expressed on the surfaces of permissive adenocarcinoma cell lines. Antibodies and small interfering RNA (siRNA) directed against Nectin-4 were capable of blocking measles virus infections in the MCF7 breast adenocarcinoma cell line. In addition, a direct virus binding assay using flow cytometry, indicated that Nectin-4 was a bona fide receptor that supported virus attachment to the host cell. We subsequently observed that both human Nectin-4 and its mouse homologue could function as receptors since this molecule is highly conserved across species. Several different laboratory and wild-type strains of measles were tested, and all were able to use Nectin-4 as a receptor.

Nectin-4/PVRL4 is a member of the poliovirus receptor-like proteins (PVRLs) which are also known as nectins. These are cell adhesion proteins of the immunoglobulin superfamily [139]. Nectin-4/PVRL4 is a 510-amino acid transmembrane protein that has a predicted molecular weight of 55.5 kDa. It migrates on sodium dodecyl sulphate (SDS) polyacrylamide gels with a molecular mass of 66 kDa due to N-glycosylation. Nectin-4 is highly expressed in embryos and has been shown to be a tumor cell marker for breast, lung, and ovarian carcinomas. It is often highly expressed in these cancers [140,141,142]. In normal tissue it is abundant in the placenta and weakly expressed in the trachea. However, in the adult mouse, Nectin-4 transcripts were also weakly detected in the testis, lung, and brain [139]. The 139-amino acid cytoplasmic domain has been shown to interact with the F-actin-associated protein afadin and both proteins colocalize at the intercellular adherens junctions of epithelial cells. Nectin-4 also interacts with its own V domain and that of Nectin-1/PVRL1, but not with other members of the nectin family. The human Nectin-4 protein shows 99% amino acid identity with the orangutan homologue, 92% identity with the mouse protein, and 95% identity with the putative canine gene product. Nectins are cell adhesion molecules that are found in the adherens junctions and normally lie just beneath the tight junctions to form a circumferential actin-associated belt around the epithelial cell. The adherens junctions are comprised of the nectins and cadherins [143]. These proteins exhibit trans-binding properties with their molecular counterparts on adjacent cells. Binding between the cadherins is calcium dependent while interaction between the nectins is not. The nectin extracellular domain is also associated with α_v_β_3_ integrin. The cytoplasmic carboxyl terminal domain of nectins binds to the protein afadin which in turn binds to actin. Cadherins interact with actin via α-catenin, β-catenin, and epithelial protein lost in neoplasm (EPLIN). In tumor cells, Nectin-4 is over-expressed on the cell surface and can be shed into the sera of patients. It has been proposed to be a marker for highly metastatic breast cancer [142,144]. Nectin-4 contributes to the metastasis, proliferation, and survival of migrating breast cancer cells [145]. As previously stated, Nectin-4 has been shown to interact in *trans* with Nectin-1/PVRL1 on adjacent cells, which triggers proliferation of the cancer cell through interaction in *cis* with V β_6_ integrin. This interaction triggers the proto-oncogene tyrosine protein kinase Src (c-Src)/Src-homology 2 domain containing phosphatase 1 (SHP-1) growth signal pathway and causes the cancer cell to proliferate [145].

The nectins are also entry receptors for other viruses. Poliovirus receptor (PVR/CD155) is the prototype member of the nectin family, and it was first discovered to be the receptor for poliovirus [146]. Nectin-1 (PVRL1) is the major receptor for herpes simplex virus (HSV). It mediates entry of all HSV-1 and HSV-2 strains as well as animal alphaherpesviruses [147]. Another nectin, Nectin-2 (PVRL2), serves as an alternative entry factor for some herpesviruses [148]. The multiple receptors enable HSV-1 and HSV-2 to enter different cell types [149]. Nectin-1 is expressed on the cell surface of many tumors and this has suggested a role for HSV in oncolytic therapy [150].

**Figure 10:**
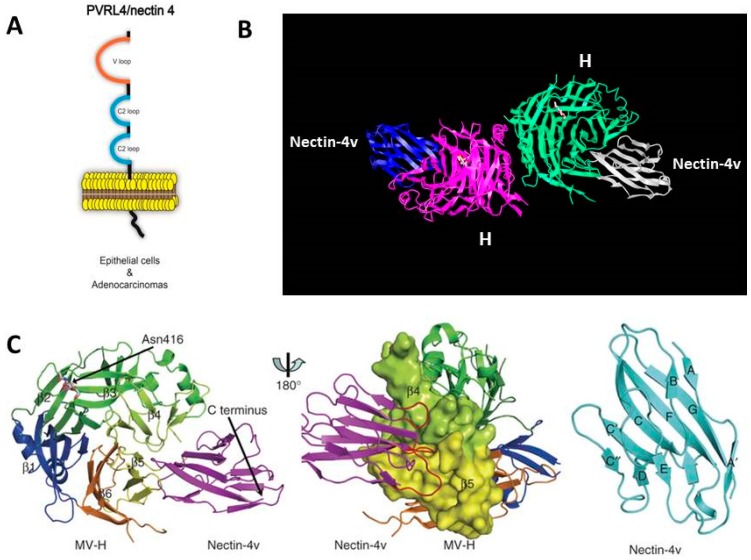
Structure of the head region from the H protein of MeV bound to the V region of Nectin-4. (**A**) Schematic of Nectin-4 showing the V and two C2 regions of the extracellular domain, the membrane spanning region, and the intracellular cytoplasmic tail; (**B**) Structure derived by X-ray crystallography showing heads of the H protein dimer interacting with the V regions of Nectin-4; (**C**) Structure of the head from β4 and β5 regions of monomeric H protein interacting with of the V domain of Nectin-4 (Nectin-4v). Adapted by permission from the Nature Publishing Group, Macmillan Publishers Ltd.: Zhang, X.; Lu, G.; Qi, J.; Li, Y.; He, Y.; Xu, X.; Shi, J.; Zhang, C.W.; Yan, J.; Gao, G.F. *Nat. Struct. Mol. Biol.* **2013**, *20*, 67–72 [151].

Within a year of identification of the MeV epithelial receptor, the molecular structure of H protein bound to the V region of nectin-4 (Nectin-4v) was reported [151]. H protein derived from wild-type strain IC-B folded into the characteristic six-bladed β-propeller with each blade comprised of a four-stranded β-sheet. The extra N-glycosylation site at position 416 of the H protein was clearly observed. The V domain adopted its characteristic immunoglobulin fold (IgV) and inserted into the concave hydrophobic side groove formed by the β4 and β5 blades (Figure 10). Site I on the V domain conferred strong stabilization through hydrophobic interactions. Residues Ser99–Phe106 of Nectin-4v interact with MeV H residues Tyr524, Leu526, Tyr541, and Tyr543 of the β5 blade on one side and Pro458, Met459, Leu462-Gly 465, Leu 482, and Phe 483 of the β4 blade. Phe101–Gly104 of Nectin-4v has a pivotal role in MeV H protein interaction. Site II involves Gln30, Gly32, and Gln33 of Nectin-4v which interact with Thr 498, Tyr499 and Asp505 in the intervening loop between blades 4 and 5 of MEV H. Finally, site III consists of His52–Tyr55 of Nectin-4v contacting H protein residues Ly387–Ly389 and Gln 391 in the β3 blade and Tyr499 and Leu500 in the β4–β5 loop. The *K_d_* binding of H with Nectin-4 is 20 nM [138].

**Figure 11:**
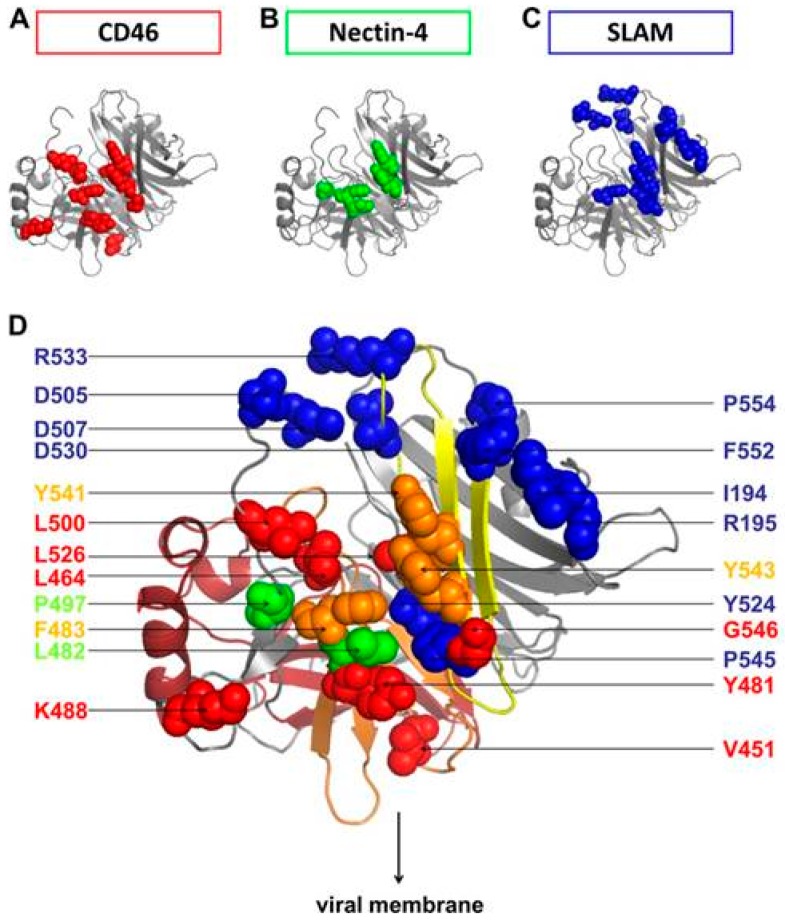
Summary of the principal residues contained in the β4, β5, and β6 regions in the head of the H protein involved in binding to its receptors. (**A**) CD46; (**B**) Nectin-4; and (**C**) SLAMF1/SLAM cellular receptors. Amino acid residues involved in binding to CD46 are shown in red, those binding to Nectin-4 in yellow, and those interacting with SLAMF1/SLAM in blue; (**D**) Overview of receptor binding residues in H protein. Overlapping interacting H residues (F483, Y541, Y543) for CD46 and Nectin-4 are shown in orange. Reprinted with permission from the American Society of Microbiology Journals: Mateo, M.; Navaratnarajah, C.K.; Syed, S.; Cattaneo, R. *J. Virol.* **2013**, *87*, 9208–9216 [152].

Using a receptor-dependent fusion assay and site-specific mutations of residues making up the putative receptor binding site on the H protein, critical residues for binding to CD46, Nectin-4, and SLAMF1 were mapped [152]. F484, Y541, and Y543 were important for binding to all three receptors. Other mutations on H were used to discriminate binding regions to the three different receptors. I194S, D530A and R533A abolished binding to SLAMF1. V451S, L464S, Y481S, K488A, T498A, and L500S reduced CD46 binding. Finally, L482S, F483S, P497S, Y541A, and Y543A blocked binding to Nectin-4. The Y543A mutation strongly reduces H binding to Nectin-4 and CD46 but has only a minimal effect on SLAMF1 binding and membrane fusion. This virus is unable to enter cells using CD46 and Nectin-4 but retains its ability to use SLAMF1 for entry. In an attempt to abrogate binding to Nectin-4 while still retaining to CD46 and SLAMF1, a L482S mutation was introduced into H. However, this change did not block Nectin-4 mediated cell entry but it did reduce cell-to-cell fusion. Based on the preceding data, Mateo et al. concluded that there was a strong overlap between the footprints of Nectin-4 and CD46 binding but not that of SLAMF1. The authors reported that Nectin-4 and CD46 interact functionally with the H protein β4–β5 hydrophobic groove, while SLAMF1 simply covers it. The authors suggested that the hydrophobic groove is not relevant for entry using SLAMF1, which interacts primarily with the β5 and β6 propeller blades of H. They also suggest that the CD46 binding site in the H protein of the measles vaccine strain may have evolved from the Nectin-4 binding site through minor changes. The results of the preceding mutational analysis of MeV H protein are summarized in Figure 11. Mutational analysis of the canonical adhesive interface of the V domain of Nectin-4 showed that the FG loop protrudes into the H-protein hydrophobic groove and that its BC loop covers the groove and helps stabilize the complex [153]. The residues D60, V62, and G63 are key residues supporting contacts by the BC loop and mutations in amino acids 61–64 of the V domain abrogate binding to H protein. A G63E substitution found in porcine Nectin-4 blocks the interaction between Nectin-4 and H protein, and helps determine species specific interaction of the virus. Recently, Takeda’s group showed that vaccine strains of MeV could use a Nectin-4 homologue in chick embryo fibroblasts as a receptor [154]. This homologue contained well-conserved FG and BC loop motifs, but the non-essential C’C’’ loop varied considerably from mammalian Nectin-4 molecules.

## 7. SLAMF1 and Nectin-4 Are Also the Receptors for Other Related Morbilliviruses

SLAMF1 was also shown to be the lymphocyte receptor for other morbilliviruses including canine distemper virus (CDV), phocine distemper, rinderpest, and peste des petits ruminants virus (PPRV) [155,156,157]. Canine distemper virus prefers to use the canine homologue of SLAMF1, and infections of cells expressing human SLAMF1 (hSLAMF1) are less efficient. There is a need for adaptation of these viruses to grow in hSLAMF1-Vero cells [158,159]. Host range expansion of CDV with adaptation to SLAMF1 species variants has been reported in carnivores [160,161]. In addition, the ability of wild type variants to CDV to adapt and infect primates has recently been reported [162,163,164].

Nectin-4 is highly conserved between different species, and functions as a receptor for morbilliviruses including canine distemper virus [165,166,167], PPRV [168,169], and phocine distemper virus [170], without the need for adaptation. As with MeV, the V domain of dog Nectin-4 mediates canine distemper virus entry and virus cell-to-cell spread [171]. Similarly, infection of epithelial cells by CDV using Nectin-4 is required for clinical disease and release of the virus, but not for immune suppression, which is mediated by interaction with SLAMF1 [167].

## 8. The Discovery of Nectin-4 Revealed a New Paradigm for Host Infection and Pathogenesis of MeV

For many years it was hypothesized that MeV initially localized to the respiratory tract and infected airway epithelial cells before disseminating through the lymphatic system and causing viremia. Major breakthroughs in our current understanding of MeV pathogenesis have unfolded over the last 10 years. A hypothetical apical airway epithelial cell receptor for MeV has been discussed at previous conferences, but its existence has not been substantiated. It is clear now that alveolar macrophages and dendritic cells that express SLAMF1 are the primary targets for MeV, which then carry MeV to the draining lymph nodes, where monocytes, T- and B-lymphocytes, that again express SLAMF1, are infected and amplify the virus, establishing primary viremia [109,116,172,173]. Once viremia is established, the virus disseminates to secondary lymphoid organs such as the thymus, spleen, appendix, and tonsils, resulting in a secondary viremia and acute immunosuppression in the host. This results in spread of the virus to distant sites within the host, including the kidneys, gastrointestinal tract, liver, and airway epithelial cells of the respiratory tract. Infection of the primary airway epithelial cells seems to occur via the basolateral surface (adherens junctions) where Nectin-4 is located, presumably through contact with infected immune cells [115,116,138]. MeV is amplified in the airway cells and released from the apical surfaces of the epithelial cells. Virus is expelled in aerosol droplets through coughing and sneezing. This new paradigm of infection has been validated through recent non-human primate infection studies using macaques [173,174]. These papers showed that a Nectin-4 “blind” recombinant MeV infected myeloid cells and lymphocytes. Infected immune cells accumulate adjacent to sub-epithelial cells of the respiratory tract, suggesting that they “seed” the in vivo infection that occurs in the airways. Only wtMeV spreads to the epithelium, to form infectious centers comprised of contiguous columnar cells.

## 9. The Receptors of Measles Virus Are Highly Expressed on Different Cancer Cells and Facilitate Viral Oncolytic Properties

MeV possesses oncolytic properties and can be used to target and infect tumor cells [175,176,177,178,179]. This was essentially a re-discovery of reports in the literature where MeV infections were shown to produce remissions of Burkitt’s lymphoma and Hodgkin’s disease [180,181,182,183,184]. Since these tumors express SLAMF1, one can postulate that wtMeV infected the tumors and elicited an immune attack against them. Many tumors express increased CD46 levels on their surface, and anti-tumor activity of Edmonston MeV has been shown in several mouse xenograft models for lymphoma, multiple myeloma, glioma, ovarian, colorectal, breast, and liver cancers [176,185,186,187,188,189,190,191]. A promising clinical trial in Zurich used Edmonston-Zagreb MeV to treat T cell lymphoma [192]. However, targeting CD46 on tumor cells may be somewhat limited, since most normal human cells express this molecule on their surface. It is also possible to re-engineer the tropism of MeV to particular tumor cell surface antigens such as carcinoma embryonic antigen (CEA), CD38, CD20, epithelial growth factor (EGF) receptor, prostate specific antigen, myeloma antigens by adding single-chain antibodies to the distal end of the H protein [177,193,194,195,196,197,198,199]. Clinical trials with EdMeV and EdMeV-sodium iodide symporter (NIS) have been initiated at the Mayo Clinic and are being used to treat multiple myeloma, ovarian cancer, glioblastoma, pancreatic cancer, and medulloblastoma [200,201,202,203]. We originally showed that many breast, lung, colon, liver, and pancreatic tumor cell lines expressed Nectin-4 and were susceptible to infection by wtMeV [133]. Our group has shown that human breast cancer xenografts in nude and non-obese diabetic/severe combined immunodeficiency (NOD/SCID) mice can be infected with MeV expressing eGFP and firefly luciferase reporter genes. Another laboratory has generated xenograft tumor models for breast, lung, and colon xenograft tumors in immune deficient mice and have shown that these tumors can be infected with SLAMF1 blind measles virus [204,205,206]. We and others have suggested that this approach could be also used to treat canine cancers [207,208]. Although MeV infection slows progression and shrinks the tumor diameter in xenograft models, the cancers are not totally eliminated. Further vector development to enhance virus replication in the tumor and promote tumor surveillance by the host immune system may improve the chances for cancer remission.

## 10. Conclusions and Future Directions

Receptor interaction determines the host specificity and tissue susceptibility of morbillivirus infections. The attachment of measles virus to CD46, SLAMF1, or Nectin-4 receptors on the host cell, using the H envelope protein, is the initial event of MeV infection, and the past 23 years have yielded a detailed understanding of the process for attachment. These interactions between receptors and the H protein have been elucidated through X-ray diffraction of crystals comprised of H complexed to portions of the receptors. Coupled with analysis by site-specific mutagenesis, the binding regions on H protein have been dissected and used to produce recombinant MeVs that are blind to 1 or 2 receptors and specific for another. This knowledge is being translated to produce better attenuated vaccines and yield more targeted approaches to viral oncolysis. Transgenic mice containing the human receptor homologues have also been generated, and these small animal models may eventually be useful for morbillivirus vaccine and antiviral drug studies.

Since the proteins representing MeV receptors also have the ability to transduce signals following ligand binding, it is conceivable that virus binding affects downstream events in the host cell. This has been a neglected field of study, but CD46 activation has been shown to influence immune cell function, cytokine production, cytoskeletal (actin) rearrangements, and autophagy [209,210]. Similarly, SLAMF1 can serve as a microbial sensor following binding to a pathogen and has been shown to stimulate cytokine production and immune cell proliferation [95]. In this regard, MeV H protein was recently shown to bind to SLAMF1, to decrease activation markers on dendritic cells and downregulate IL-12 [211]. There was also one abstract from the 15^th^ International Negative Strand Virus Meeting (2015) that has implicated SLAMF1 in the uptake of MeV through macropinocytosis [212]. Finally, Nectin-4 activation has been shown to stimulate cell proliferation and survival. Its involvement in growth factor and endocytotic pathways (through Rac1 and p21 involvement) has been reported [141,145]. In general, signal transduction pathways that are associated with the three receptors could be important for uptake and survival of MeV in the host cell, but more research will be required to confirm this hypothesis.

MeV and other members of the morbillivirus family have the potential to mutate through the action of their RNA polymerase, but genetic variation is highly constrained for both the H and F proteins [38,213]. This may be due to specific mechanical needs and constraints on the viral membrane proteins during attachment, activation of fusion, and merging of the two membranes. Viruses containing mutations that are not compatible with these processes would not be expected to survive. The receptor binding regions of MeV H protein are highly conserved and there is little variation in amino acid sequences in both laboratory/vaccine and wild-type MeV strains [38,39,41,214,215]. Furthermore, wtMeV and vaccine/laboratory strains have a higher affinity for SLAMF1 compared to CD46, and wtMeV only adapts to use CD46 out of necessity, when SLAMF1 and Nectin-4 are not available. However, some strains of wtMeV have been reported to weakly bind to CD46 on immune peripheral blood mononuclear cells, suggesting that CD46 is an auxiliary latent receptor waiting for an opportunity to serve [216]. A detailed investigation of persistent infections and potential changes of receptor usage within the host over long periods has not been reported. Such a scenario could reveal additional receptors, perhaps those postulated for the brain, and the generation of neurotropic virus variants [217]. Other morbilliviruses also have the ability to mutate somewhat, since the H protein from CDV must adapt to use human SLAMF1, but it only requires one amino acid change to do so (D540G). These changes compensate for two amino acid differences (L70P) and E71G) in dog SLAMF1. On the other hand, Nectin-4 is highly conserved among mammalian species and there is no need for adaptation by other members of the morbillivirus family and the Vero-hNectin-4 (hPVRL4) cell line has been used to isolate PPRV, phocine distemper, canine distemper, and feline distemper viruses. Further investigations using animal models with persistent viral infections in other organs could yield intriguing results with regards to receptor usage in the future.

In addition to CD46, SLAMF1, and Nectin-4, the existence of neurological receptors for both MeV and CDV on neurons and astrocytes have been hypothesized [218,219,220]. One laboratory has claimed that Nectin-4 is expressed in the brain and contributes to neuron infections [166]. However, another laboratory has demonstrated only weak and inconsistent punctate staining for Nectin-4 in the brainstem neurons. Nectin-4 expression could be seen in the ependymal cell layer of the medulla oblongata, with only minor staining of the meninges. Primary astrocytes and white matter of the dog brain were shown to be completely devoid of Nectin-4 [218]. Subsequently, SLAMF1 and Nectin-4 independent infections of astrocytes were reported using a neurological strain of CDV containing the red fluorescent protein reporter [218]. Infections of primary astrocytes derived from the canine brain were evident between 0 and 16 days of infection, and the virus was transmitted from cell to cell. Infections of neurons are less clear and they have been suggested to occur through direct interaction with the F protein or through synaptosomal transmission [220].

The process where H binds to its receptor and triggers F is just beginning to be understood [13,45]. The binding of H protein to its receptor causes a conformational change in the adjacent F protein, initiates fusion between the virus and host cell membranes. However, a stabilized headless H protein stalk is able to trigger membrane fusion [221]. This is hypothesized to occur through an interaction of a spacer (amino acids 126–133) on the H stalk. Prior to receptor attachment, it is believed that a head–stalk interaction in the H protein blocks the interaction of spacer with the F protein trigger, stabilizing H in an auto-repressed state. Receptor contact disrupts the head to spacer interaction and unlocks the stalk, allowing it to rearrange and trigger the conformational change in F [46]. These protein interactions are also important for syncytia formation and spread of the virus from cell to cell. A more detailed molecular view of this process is likely to emerge in the future.

The interaction of MeV with its receptors has had broad implications in understanding the pathogenesis of this virus. This knowledge has helped us to understand the immune suppressive properties of the virus. It has allowed us to predict the pathway of infection from immune cells into epithelial cells, including those of the airways, intestines, and bladder, where virus is subsequently amplified. MeV is then expelled into the environment by coughing and sneezing and is recognized to be one of the most contagious human pathogens. Finally, the receptors have helped us to discover the oncolytic properties of this virus, which is leading to the treatment and cure of a variety of cancers. In the future, other receptors for MeV on different cell types may also be found, and lead to further interesting applications.
